# Inhibitors of Fatty Acid Synthesis Induce PPAR**α**-Regulated Fatty Acid **β**-Oxidative Genes: Synergistic Roles of L-FABP and Glucose

**DOI:** 10.1155/2013/865604

**Published:** 2013-02-26

**Authors:** Huan Huang, Avery L. McIntosh, Gregory G. Martin, Anca D. Petrescu, Kerstin K. Landrock, Danilo Landrock, Ann B. Kier, Friedhelm Schroeder

**Affiliations:** ^1^Department of Physiology and Pharmacology, Texas A&M University, TAMU 4466, College Station, TX 77843-4466, USA; ^2^Department of Pathobiology, Texas A&M University, TAMU 4467, College Station, TX 77843-4467, USA

## Abstract

While TOFA (acetyl CoA carboxylase inhibitor) and C75 (fatty acid synthase inhibitor) prevent lipid accumulation by inhibiting fatty acid synthesis, the mechanism of action is not simply accounted for by inhibition of the enzymes alone. Liver fatty acid binding protein (L-FABP), a mediator of long chain fatty acid signaling to peroxisome proliferator-activated receptor-**α** (PPAR**α**) in the nucleus, was found to bind TOFA and its activated CoA thioester, TOFyl-CoA, with high affinity while binding C75 and C75-CoA with lower affinity. Binding of TOFA and C75-CoA significantly altered L-FABP secondary structure. High (20 mM) but not physiological (6 mM) glucose conferred on both TOFA and C75 the ability to induce PPAR**α** transcription of the fatty acid **β**-oxidative enzymes CPT1A, CPT2, and ACOX1 in cultured primary hepatocytes from wild-type (WT) mice. However, L-FABP gene ablation abolished the effects of TOFA and C75 in the context of high glucose. These effects were not associated with an increased cellular level of unesterified fatty acids but rather by increased intracellular glucose. These findings suggested that L-FABP may function as an intracellular fatty acid synthesis inhibitor binding protein facilitating TOFA and C75-mediated induction of PPAR**α** in the context of high glucose at levels similar to those in uncontrolled diabetes.

## 1. Introduction

Obesity and overweight are worldwide health problems, affecting >50% of the US population and exceeding tobacco as the major cause of preventable mortality in the USA [1–3]. Obesity is associated with the development of type 2 diabetes (NIDDM), cardiovascular disease, nonalcoholic fatty liver disease (NAFLD), and increased mortality [2, 4–7]. Consequently, increasing effort in therapeutics has focused on the development of drugs such as TOFA, C75, and cerulenin that target the fatty acid metabolic pathway to inhibit synthesis. C75 is a competitive irreversible, slow-binding inhibitor of fatty acid synthase (FASN), cerulenin is suicide inhibitor of FASN, and TOFA is an allosteric inhibitor of acetyl CoA carboxylase (ACC) [8–11]. While these agents lower whole body and adipose tissue weight, their mechanism(s) of action is not simply accounted for by inhibition of the FASN and ACC enzymes alone.

Increased malonyl-CoA, for example, inhibits carnitine palmitoyl transferase 1A (CPT1A, the rate limiting enzyme in mitochondrial fatty acid *β*-oxidation) [8, 11]. Since the ACC inhibitor TOFA decreases malonyl CoA while the two FASN inhibitors (C75, cerulenin) increase malonyl-CoA, it was anticipated that TOFA would enhance while C75 and cerulenin would inhibit CPT1A and fatty acid *β*-oxidation [8, 11, 12]. Despite these opposite expectations, however, both TOFA and C75 enhanced CPT1A activity and fatty acid *β*-oxidation while cerulenin exhibited a biphasic effect characterized by short-term (1 h) inhibition followed by longer term (3–5 h) stimulation fatty acid *β*-oxidation [8, 11–13]. Similar opposite effects of closely related fatty acid synthesis inhibitors on fatty acid oxidation are not uncommon [14]. While some paradoxical effects of these agents have been attributed in part to actions in the central nervous system (e.g., reduced food intake), this alone also does not completely explain the effects of TOFA (does not inhibit feeding), C75 (inhibits feeding), and cerulenin (activity not highly correlated with food intake) on increasing CPT1A and fatty acid *β*-oxidation [1–3, 8, 10, 13].

Recent studies from our laboratory and other laboratories have established a signaling pathway, whereby the liver fatty acid binding protein (L-FABP) facilitates uptake and intracellular targeting of poorly soluble fatty acids and fibrates to PPAR*α* in the nucleus (review in [16–18]). PPAR*α* binds and is activated by LCFA and LCFA-CoA and a variety of lipid lowering drugs (fibrates, statins) [19–26]. Ligand activation of PPAR*α* induces transcription of many proteins and enzymes involved in fatty acid uptake (membrane fatty acid transporters (FATPs), liver fatty acid binding protein (L-FABP)), intracellular fatty acid transport (L-FABP), and fatty acid oxidation (L-FABP, CPT1A, CPT2, ACOX1) (review in [17, 27–29]). Unlike other members of the fatty acid binding protein family, L-FABP is unique in its broad specificity for lipidic ligands, binding not only LCFA and LCFA-CoA, but also a variety of therapeutic agents such as fibrates and their CoA thioesters (review in [16–18, 30–33]).

**Figure 1:**
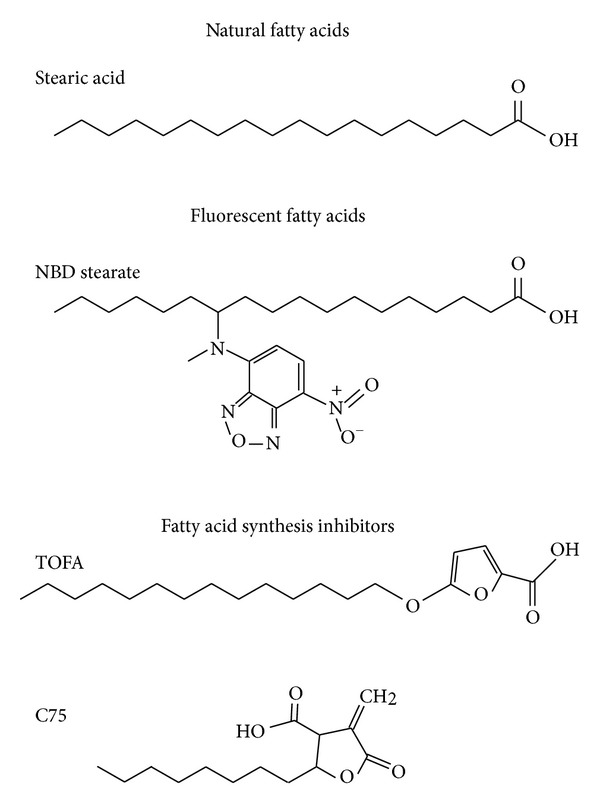
Structural comparisons of the fatty acid synthesis inhibitors TOFA and C75 with natural and fluorescent fatty acids.

Because of their structural resemblance to fatty acids (Figure 1), we postulated that some of the fatty acid synthesis inhibitors (esp. TOFA, C75) may also be bounded by L-FABP and targeted to induce PPAR*α* transcription of fatty acid *β*-oxidative enzymes in mitochondria (CPT1A, rate limiting; CPT2) and peroxisomes (ACOX1, rate limiting). The focus of this study was to examine the extent to which (i) L-FABP binds fatty acid synthesis inhibitors; (ii) fatty acid synthesis inhibitors induce PPAR*α* transcription of fatty acid *β*-oxidative enzymes (CPT1A, CPT2, ACOX1); (iii) L-FABP facilitates fatty acid synthesis inhibitor action on PPAR*α*; (iv) high glucose impacts L-FABP mediated fatty acid synthesis inhibitor signaling to PPAR*α*. These issues were addressed using purified L-FABP, fluorescent ligand binding assays, circular dichroism (CD), and cultured primary hepatocytes from wild-type and L-FABP (−/−) null mice.

## 2. Materials and Methods

### 2.1. Materials

Recombinant liver fatty acid binding protein (L-FABP) and sterol carrier protein-2 (SCP-2) were prepared as described [34, 35]. Albumin fraction V, fatty acid free (10% solution for tissue culture), TOFA (5-tetradecyloxy-2-furancarboxylic acid), C75 (4-methylene-2-octyl-5-oxotetrahydrofuran-3-carboxylic acid), oleic acid, oleoyl-CoA, D (+) glucose, dexamethasone, insulin, and acyl CoA synthase from *Pseudomonas* sp. were purchased from Sigma (St. Louis, MO, USA). NBD stearate (12-(N-methyl)-N-[(7-nitrobenz-2-oxa-1,3-diazol-4-yl)-amino]-octadecanoic acid) was purchased from Avanti Polar Lipids (Alabaster, Alabama, USA). ANS (aminonaptholsulfonic acid) was from Cayman Chemical Company (Ann Arbor, MI, USA). Collagenase B was from Roche, (Life technologies, Carlsbad, CA, USA). Dulbecco's modified Eagle medium DMEM/F12, glucose-free DMEM, fetal bovine serum, gentamycin, and Hank's balanced salt solution free of calcium and magnesium (HBSS) were obtained from Gibco/Invitrogen (by Life Technologies, Carlsbad, CA, USA). RN-ase-free DNase set and RN-easy kit were obtained from Qiagen GmbH (Hilden, Germany) and Qiagen Sciences (Maryland, USA), respectively. TaqMan, One-Step RT-PCR Master Mix reagents, and TaqMan Gene Expression Assays for CPT1A (carnitine-palmitoyl-transferase 1 A), CPT2 (carnitine-palmitoyl-transferase 2), and ACOX1 (acyl-coenzyme A oxidase 1) were from Applied Biosystems (by Life Technologies, Carlsbad, CA, USA). Rabbit polyclonal antibodies against rat liver fatty acid binding protein (L-FABP), human sterol carrier protein-2 (SCP-2), and mouse acyl CoA binding protein (ACBP) were prepared as described in [36–38]. Rabbit polyclonal antibodies to liver X receptor-*α* (LXR*α*, sc-1201) and sterol response element binding protein-1 (SREBP1, sc-367) were obtained from Santa Cruz Biotechnology Inc. (Santa Cruz, CA, USA). Rabbit polyclonal antibody to carbohydrate response element binding protein (ChREBP, ab81958) was purchased from Abcam PLC (Cambridge, MA, USA). Rabbit polyclonal antibody to PPAR*α* (PA1-822A) was from Pierce Antibodies (Rockford, IL, USA).

#### 2.1.1. Synthesis, Purification, and Characterization of TOFyl-CoA and C75-CoA

The active forms of TOFA and C75 are the respective CoA thioesters, which accumulate within the cell and are not or only slowly metabolized [10, 12, 39, 40]. Determination of whether L-FABP and SCP-2 interacted with the fatty acid synthesis inhibitors and/or their CoA thioesters required the synthesis of the respective noncommercially available CoA thioesters. TOFyl-CoA was prepared by chemical synthesis [41]. Since C75-CoA prepared by chemical synthesis [41] yields a chemical structure very different from that obtained enzymatically *in vivo*, the C75-CoA was prepared by enzymatic synthesis with long chain acyl CoA synthase as described in [39, 40]. TOFyl-CoA and C75-CoA were purified by HPLC as previously described [15]. The formation of thioester bond was confirmed by disappearance of the CoA derivatives and appearance of free CoA HPLC peaks upon alkaline hydrolysis. UV absorbance spectra were obtained with Cary 100 Scan UV-Visible Spectrophotometer (Varian, Inc., Palo Alto, CA, USA). To confirm that the CoA derivatives had the correct molecular weight, TOFyl- and C75-CoA products were also examined by matrix-assisted laser desorption ionization time-of-flight (MALDI-TOF) mass spectrometry utilizing a Shimadzu/Kratos Axima CFR MALDI-TOF mass spectrometer (Columbia, MD, USA) in reflectron mode by the Protein Chemistry Laboratory (Texas A&M University, College Station, TX, USA). Samples were analyzed by the dried-drop method using *α*-4-hydroxycinnamic acid (Sigma-Aldrich) as the matrix. The instrument was calibrated with angiotensin (*m*/*z* = 1046.5) and fibrinopeptide B (*m*/*z* = 1570.7). The calibrants were obtained from Sigma-Aldrich. For each sample, the additional labeled peaks corresponded to the parent ion plus one, two, three, or four potassium ions.

### 2.2. Ligand Binding Assays

#### 2.2.1. Fluorescent NBD-Stearic Acid Binding to L-FABP and SCP-2

The binding constants of NBD stearate to L-FABP and SCP-2 were obtained by titrating a 2 mL sample of L-FABP (25 nM) or SCP-2 (25 nM) in 10 mM phosphate buffer (pH 7.4) with small increments of NBD stearate at 24°C. NBD stearate fluorescence emission spectra (515–600 nm) were recorded using a Varian Cary Eclipse Fluorescence Spectrophotometer (Varian, Inc., Palo Alto, CA, USA), with 490 nm excitation. The binding curves were constructed by plotting NBD stearate fluorescence intensity increase (*F* − *F*_0_) versus concentration [NBD stearate], with *F* being fluorescence intensity of NBD stearate in the presence of proteins (at 530 nm for binding to SCP-2 and at 548 nm for binding to L-FABP) and *F*_0_ being NBD stearate fluorescence intensity in buffer (at the same wavelength as for *F*). Curve fitting of the binding curve yielded *F*_max⁡_, the maximum fluorescence intensity. The dissociation constant *K*_*d*_ was calculated from the slope of double reciprocal plots 1/[1 − (*F* − *F*_0_)/*F*_max⁡_] versus [NBD-stearate]/[(*F* − *F*_0_)/*F*_max⁡_] as described [20, 42].

#### 2.2.2. Displacement of L-FABP or SCP-2-Bound Fluorescent Ligands: NBD Stearate and ANS

Advantage was taken of the fact that NBD-stearic acid bound to L-FABP (two ligand binding sites) as well as SCP-2 (single-ligand binding site) to design a NBD-stearic acid displacement assay that allowed determination of which fatty acid synthesis inhibitors or their CoA thioesters bound to these LCFA/LCFA-CoA binding proteins [20, 42]. Displacement of NBD-stearate from L-FABP and SCP-2 was determined by incubating L-FABP or SCP-2 (25 nM in 10 mM phosphate buffer) with NBD stearate (40 nM) for 12 min to obtain maximal fluorescence, followed by titration with increasing amount of ligand. From the displacement curve, the *K*_*i*_ value was calculated as described [20, 42]. All experiments were carried out with a thermostated cuvette to maintain temperature at 24°C using a circulating water bath. ANS displacement assay was performed as described [30, 33].

#### 2.2.3. Tyrosine Fluorescence Quenching

Tyrosine fluorescence of L-FABP (100 nM) in 10 mM phosphate buffer was monitored by scanning from 290 to 400 nm, with excitation wavelength 280 nm, before and after small increments of added binding ligand. The binding curve was constructed by plotting tyrosine fluorescence intensity at 305 nm versus concentration of the ligand. *K*_*d*_ was then calculated as described [19, 20].

### 2.3. Secondary Structure Determination by Circular Dichroism (CD) 

Circular dichroism (CD) spectra were obtained utilizing a JASCO J-815 CD spectrometer (JASCO Analytical Instruments, Easton, MD, USA). Each sample for CD analysis contained recombinant rat L-FABP (1 *μ*M) in 10 mM potassium phosphate (pH 7.4) with or without ligand (10 *μ*M). Samples were scanned from 190 to 250 nm using the following conditions: step resolution, 1 nm; bandwidth, 2 nm; sensitivity, 10 mdeg; scan rate, 50 nm/min; and time constant, 1 s. For each measurement, 10 scans were averaged, background-subtracted, smoothed using the Means-Movement smoothing method (convolution width, 5) and converted to mean residue molar ellipticity utilizing the Spectra Manager Version 2 software as supplied by the instrument manufacturer (Spectra Analysis Version 2.03.04, Build 5). The percentage composition of *α*-helices (regular, distorted, total), *β*-sheets (regular, distorted, total), turns, and unordered structures was determined utilizing the CDPro program as supplied by the instrument manufacturer using the SDP48 reference set (soluble + denatured protein) [20, 34]. This program allows the percentage calculation of various secondary structures by three different methods: CDSSTR, CONTIN, and SELCON3. Statistical analysis was performed by one-way analysis of variance (ANOVA) combined with the Newman-Keuls multiple-comparisons posttest (GraphPad Prism Version 3.03, San Diego, CA, USA).

### 2.4. Wild-Type and L-FABP Gene-Ablated Mice

Wild-type mice on a C57BL/6N background purchased from Charles River Laboratories (Wilmington, MA, USA) were obtained from the National Cancer Institute (Frederick Cancer Research and Development Center, Frederick, MD, USA). L-FABP (−/−) null mice were obtained by targeted disruption of the L-FABP gene [36] and backcrossed to C57Bl/6N background to the N10 (99.9% homogeneity) generation. For hepatocyte isolation, livers were collected from male mice aged 3–6 mo. Mouse protocols were approved by the Institutional Animal Care and Use Committee (IACUC) at Texas A&M University. Mice were kept under a constant 12/12 light-dark cycle and had access to food and water *ad libitum*.

### 2.5. Mouse Primary Hepatocyte Isolation and Culture

Mouse hepatocytes were isolated from livers of male wild-type and L-FABP null mice as described [43, 44]. To study the effect of TOFA and C75 at different glucose levels, mouse hepatocytes were cultured overnight with DMEM/F-12 supplemented with 5% FBS. Cells were incubated with glucose-free DMEM supplemented with fatty acid-free BSA (40 *μ*M), 100 nM insulin, 10 nM dexamethasone, and without (no inhibitor) or with the *de novo* fatty acid synthesis inhibitors C75 or TOFA (10 *μ*g/mL) for 30 min (1 mL/well). Cells were incubated further for 5 hr with 6 or 20 mM glucose without or with the inhibitors.

### 2.6. Hepatocyte mRNA and Western Blotting Measurements 

Transcription of key enzymes in mitochondrial (CPT1A and CPT2) and peroxisomal (ACOX1) *β*-oxidation of long chain fatty acid (LCFA) was determined by rtPCR similarly as described [44]. Briefly, the RNeasy mini kit from Qiagen Sciences (MD, USA) was used as per the manufacturer's instructions to isolate total RNA, which was then quantified spectrophotometrically. Quantitative real-time PCR was performed with an ABI PRISM 7000 Sequence Detection System (SDS) from Applied Biosystems (Foster City, CA, USA) to determine relative mRNA expression for CPT1A, CPT2, and ACOX1. The thermal protocol was as follows: 48°C for 30 min, 95°C for 10 min before the first cycle, 95°C for 15 sec, and 60°C for 60 sec, repeated 40 times. TaqMan One-Step Master Mix and Gene Expression Assays for mouse CPT1A (Mm 00550438_m1), CPT2 (Mm 00487202_m1), and ACOX1 (Mm 00443579_m1) were purchased from Applied Biosystems (Life Technologies, Carlsbad, CA, USA). Triplicates of each sample were analyzed with ABI PRISM 7000 SDS software (Applied Biosystems) to determine ΔCt relative to a positive control (18S housekeeping gene). The fold change in the abundance of CPT1A, CPT2, and ACOX1 mRNAs was determined in primary mouse hepatocytes treated with albumin (40 *μ*M) in the absence and presence of lipid synthesis inhibitors (C75, TOFA, 10 *μ*g/mL) in glucose-free DMEM medium supplemented with 100 nM insulin, 10 nM dexamethasone, and either 6 or 20 mM glucose. The comparative 2^−ΔΔCt^ calculation method was used as described in User Bulletin 2, ABI Prism 7000 SDS (Applied Biosystems), and earlier [45]. Finally, protein levels of liver fatty acid binding protein (L-FABP), sterol carrier protein-2 (SCP-2), acyl CoA binding protein (ACBP), peroxisome proliferator activated receptor (PPAR*α*), liver X receptor-*α* (LXR*α*), carbohydrate response element binding protein (ChREBP), and sterol response element binding protein (SREBP) were determined by western blotting as described [44].

### 2.7. Hepatocyte Cytosolic Glucose Level Measurements 

Hepatocytes were plated 2 × 10^5^ per well in 12-well tissue culture plates (Becton Dickinson and Company, Franklin Lakes, NJ, USA). After incubation with culture medium containing TOFA or C75 with increasing glucose level as described previously, hepatocytes were washed quickly with a cold solution of MgCl_2_ (100 mM) with 0.1 mM phloretin [46]. Cells were scraped from the dishes with PBS plus protease inhibitor, and cells were broken open with a probe sonicator (Sonic Dismembrator 550, Fisher Scientific, Waltham, MA, USA). Samples were sedimented at 10,000 g, 4°C for 20 min. The supernatant was used for glucose analysis with the Amplite Glucose Quantitation Kit (AAT Bioquest, Inc. Sunnyvale, CA, USA) as instructed by the manufacturer. The number of cultured primary hepatocytes in each sample was calculated based on amount of protein (Bradford Assay, Sigma, St. Louis, MO, USA) compared with reference samples with a known number of hepatocytes. Cytosolic glucose concentration was calculated using 7.4 × 10^−12^ liter/cell as volume of the hepatocytes [47].

### 2.8. Hepatocyte Unesterified (Free) LCFA Determination

#### 2.8.1. Lipid Extraction

Cultured mouse hepatocytes were plated 4 × 10^5^ cells per well in 12-well tissue culture plates. After incubation with TOFA or C75 at different glucose levels as described previously, hepatocytes were washed with cold PBS 4x, scraped with cold PBS (with protease inhibitor), and homogenized with a probe sonicator (Sonic Dismembrator 550, Fisher Scientific, Waltham, MA, USA). A 20 *μ*L sample was saved for protein analysis using the Bio-Rad protein assay reagent, (Bio-Rad, Hercules, CA, USA). Samples were extracted twice with 1% Triton X-100 in pure chloroform (OmniSolv High purity solvent, 99.9% min. EMD Millipore, Billerica, MA, USA). The extract was centrifuged for 5–10 minutes at top speed in a refrigerated microcentrifuge. The organic phase (lower phase) was collected, dried with a stream of N_2_, and dried *in vacuo* for another 30 min to remove chloroform.

#### 2.8.2. Unesterified (Free) LCFA Assay

The LCFA content of each sample was measured with the Free Fatty Acid Quantification Kit from BioVision, Inc. (Mountain View, California, USA) according to manufacturer's instructions, using a standard of palmitic acid included in the kit. The dried lipids (in Triton X-100) were dissolved in 200 *μ*L of Fatty Acid Assay Buffer by vortexing extensively for 5 min followed by assay of triplicate 50 *μ*L aliquots of the extracted sample.

## 3. Results

*Structural Similarity of Fatty Acid Synthesis Inhibitors to Naturally Occurring Long Chain Fatty Acids (LCFAs) and Fluorescent LCFA Analogues*

The structure of the fatty acid synthesis antagonist TOFA has significant similarity to LCFAs such as the naturally occurring stearic acid and fluorescent NBD-stearic acid, as evidenced by a long, hydrophobic acyl chain and a carboxyl terminus with similar total length (Figure 1). While the fatty acid synthase (FASN) inhibitor C75 also has a carboxyl terminus, the chain length is much shorter (11 carbons) than that of stearic acid or TOFA (Figure 1). Therefore, potential binding of TOFA and C75 to L-FABP and SCP-2 was examined using fluorescent binding assays not requiring separation of bound from free ligand as described in Methods. Since the active forms of TOFA and C75 are thought to be the respective CoA thioesters, binding assays were performed with L-FABP because it binds both LCFA and LCFA-CoA, trafficks to and binds PPAR*α* within the nucleus, and enhances LCFA and LCFA-CoA transport into nuclei (review in [16, 17, 48–50]). SCP-2 was used as a control because it binds both LCFA and LCFA-CoA but is not significantly distributed to nuclei or interact with PPAR*α* (review in [16, 17, 48–50]).

*NBD-Stearic Acid Binds with Higher Affinity to L-FABP than SCP-2*

**Figure 2:**
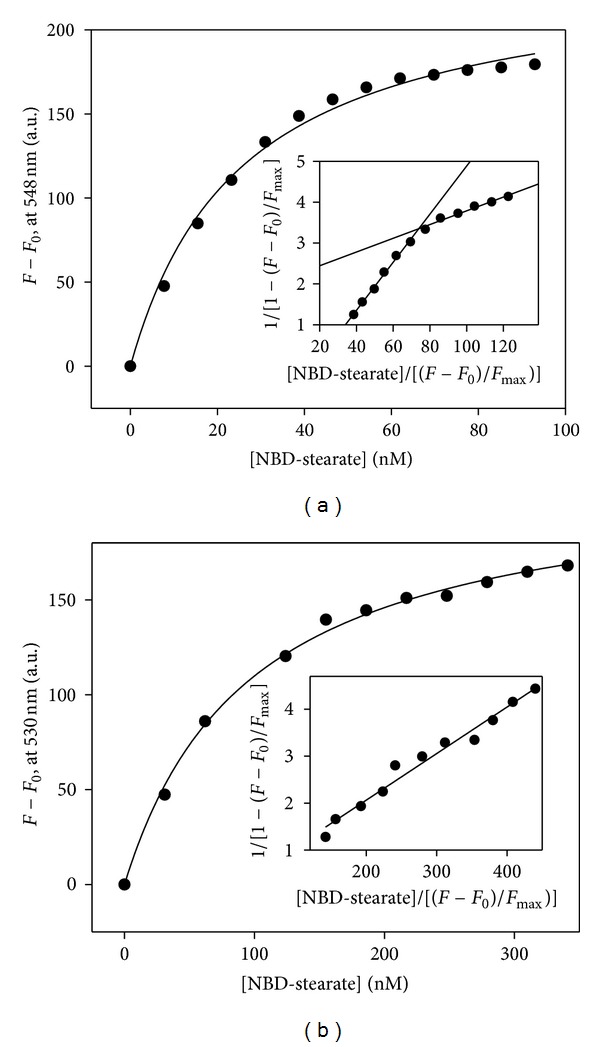
Representative binding curves of stearic acid binding to L-FABP (panel a) and SCP2 (panel b). NBD-stearic acid binding curves were obtained as described in Methods. Briefly, a 2 mL sample of L-FABP (25 nM) or SCP-2 (25 nM) in 10 mM phosphate buffer (pH 7.4) was titrated with small increments of NBD stearate at 24°C (Methods). NBD-stearate fluorescence emission spectra (515–600 nm) were recorded with 490 nm excitation. *F*: fluorescence intensity of NBD stearate in the presence of proteins (at 530 nm for binding to SCP-2 and at 548 nm for binding to L-FABP) and *F*_0_ being NBD-stearate fluorescence intensity in buffer (at the same wavelength as for *F*). Insets are double reciprocal plots of the fluorescence binding data in the same panel.

NBD-stearic acid, a fluorescent poorly metabolizable analogue of the natural LCFA stearic acid (Figure 1) directly monitored binding to L-FABP and SCP-2 as in Methods. While NBD-stearic acid fluoresces weakly in aqueous buffer, emission increases dramatically when being bound to the ligand binding sites of L-FABP or SCP-2 (Figure 2). With increasing NBD-stearic acid, fluorescence of L-FABP- (Figure 2(a)) and SCP-2 (Figure 2(b)) bound NBD-stearic acid increased towards saturation. Double reciprocal analysis of the binding curves showed that L-FABP has two binding sites (Figure 2(a), inset), while SCP-2 has a single NBD-stearic acid binding site (Figure 2(b), inset). Analysis of multiple binding curves allowed quantitative determination of L-FABP's and SCP-2's binding characteristics to NBD-stearic acid. The high affinity L-FABP binding site (*K*_*d*1_ = 0.017 *μ*M) was nearly 4-fold higher affinity than the lower affinity binding site (*K*_*d*2_ = 0.055 *μ*M), while SCP-2 bound NBD-stearic acid with a somewhat weaker affinity near 0.1 *μ*M (Table 1).

*The Fatty Acid Synthesis Inhibitor TOFA, and Less So C75, Displaced Bound Fluorescent Ligands from L-FABP and SCP-2*

**Figure 3:**
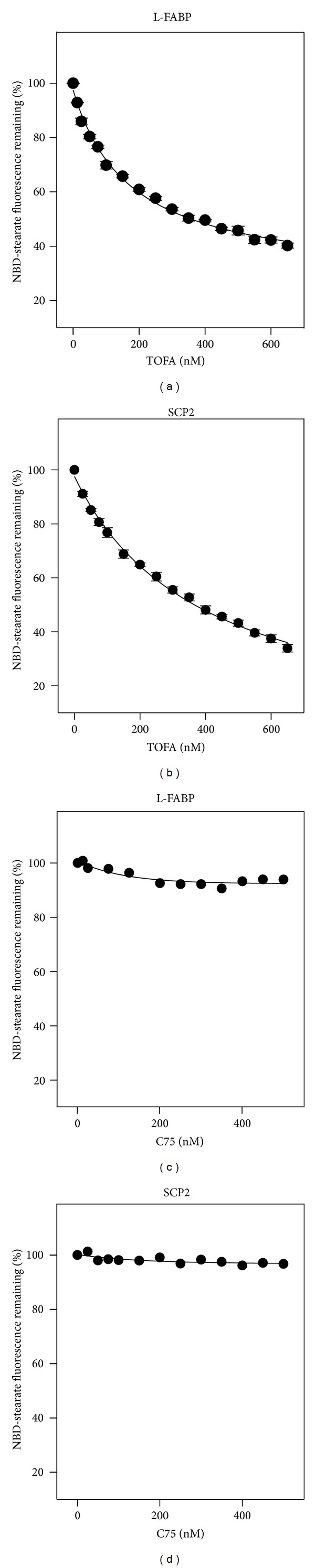
Displacement of L-FABP- and SCP2-bound NBD-stearic acid by TOFA (panels a and b) and C75 (panels c and d). NBD-stearic acid displacement assays were performed as shown in Methods. L-FABP or SCP-2 (25 nM in 10 mM phosphate buffer) was incubated with NBD stearate (40 nM) for 12 min at 24°C to obtain maximal fluorescence, then titrated with increasing amount of ligand. Mean ± SEM, *n* = 3.

A NBD-stearic acid displacement assay (Methods) revealed that TOFA significantly displaced L-FABP bound NBD-stearic acid, as shown by fluorescence decreasing to 40% at 650 nM TOFA (Figure 3(a)). A *K*_*i*_ = 0.066 *μ*M was determined for TOFA displacing NBD-stearic acid from L-FABP (Table 1). Comparison with the *K*_*d*_s of the high and low affinity NBD-stearic acid binding sites of L-FABP indicated that both stearic acid and TOFA preferentially displaced NBD-stearic acid from the weaker affinity LCFA binding site (Table 1). TOFA also displaced NBD-stearic acid bound to SCP-2 (Figure 3) with a *K*_*i*_ = 227 ± 20 nM, about half of the affinity for NBD-stearic acid binding to SCP-2 (Table 1). Thus, the *K*_*i*_s for TOFA displacing NBD-stearic acid from L-FABP and SCP-2 were in the same range as those for endogenous LCFAs such as stearic acid (Table 1).

In contrast, C75 displacement of NBD-stearic acid bound to L-FABP was weaker (Figure 3(c)) and barely detectable from SCP-2 (Figure 3(d)). Since the C75 more weakly displaced a strongly bound ligand such as NBD-stearic acid, displacement was also measured with the weaker affinity fluorescent ligand aminonaptholsulfonic acid (ANS). L-FABP bound ANS, a ligand significantly larger than NBD-stearic acid, at only a single site with *K*_*d*_s of 1.96 ± 0.09 *μ*M, consistent with earlier findings [30, 31]. C75 displaced ANS from L-FABP with a *K*_*i*_ of 5.59 ± 0.31 *μ*M, thereby confirming weaker binding than observed with the NBD-stearic acid displacement assay.

Taken together, these findings suggested that TOFA was a good high-affinity ligand for L-FABP and somewhat less than so for SCP-2. In contrast, C75 was a weaker ligand for L-FABP, and binding to SCP-2 was barely detectable.

*Synthesis, Purification, and Characterization of TOFyl-CoA and C75-CoA*

**Figure 4:**
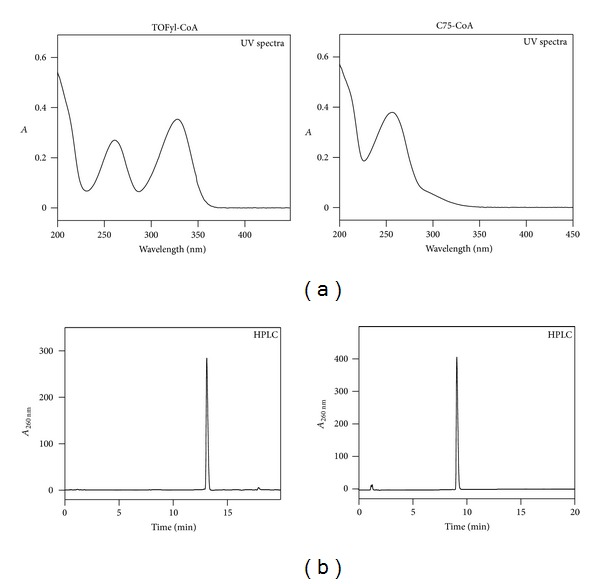
UV spectra and HPLC analysis of TOFA-CoA and C75-CoA. Ultraviolet spectra of TOFyl-CoA and C75-CoA were obtained with a Cary 100 Scan UV-Visible Spectrophotometer (Varian, Inc., Palo Alto, CA, USA) as described in Methods. TOFyl-CoA and C75-CoA were purified by HPLC as previously described [15]. When the final purified TOFyl-CoA and C75-CoA were reapplied to the HPLC column, representative HPLC runs detected only single absorbance peaks at 260 nm for TOFyl-CoA and C75-CoA with retention times of 13 and 9 min, respectively.

**Figure 5:**
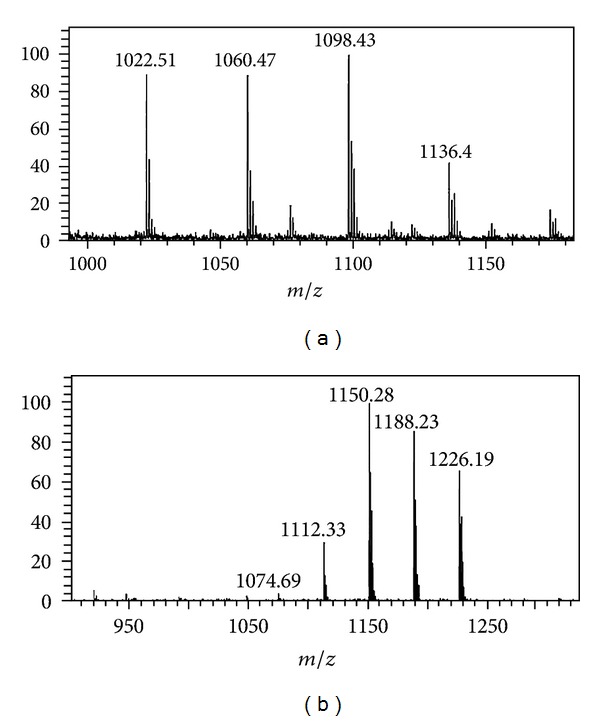
Mass spectral characterization of CoA derivatives of TOFA and C75. HPLC purified TOFYL-CoA (Panel a, *m*/*z* = 1074.69) and C75-CoA (Panel b, *m*/*z* = 1022.51) were examined by matrix-assisted laser desorption ionization time-of-flight (MALDI-TOF) mass spectrometry utilizing a Shimadzu/Kratos Axima CFR MALDI-TOF mass spectrometer (Columbia, MD, USA) in reflectron mode. Samples were analyzed by the dried-drop method using *α*-4-hydroxycinnamic acid (Sigma-Aldrich) as the matrix. The instrument was calibrated with angiotensin (*m*/*z* = 1046.5) and fibrinopeptide B (*m*/*z* = 1570.7). The calibrants were obtained from Sigma-Aldrich. The parent ions for TOFyl-CoA and C75-CoA were obtained at *m*/*z* = 1022.51 (a) and *m*/*z* = 1074.69 (b), respectively.

Since the active forms of TOFA and C75 within living cells are thought to be the respective CoA thioesters, it was important to also determine if L-FABP and/or SCP-2 bound the respective CoA thioesters [12, 39, 40]. However, TOFyl-CoA and C75-CoA are not commercially available. Therefore, TOFyl-CoA (chemical synthesis) and C75-CoA (chemical and enzymatic synthesis) were prepared and purified by HPLC as described in Methods. Formation of the respective thioester bond and purity were confirmed by (1) UV absorbance spectra detecting the thioester bonds near 260 nm for TOFyl-CoA and C75-CoA (Figure 4(a)); (2) detection of single-absorbance peaks at 260 nm with retention times near 13 and 9 min for TOFyl-CoA and C75-CoA in HPLC chromatograms (Figure 4(b)); and (3) disappearance of the CoA derivatives and appearance of free CoA HPLC peaks at earlier retention times upon alkaline hydrolysis (not shown). Matrix-assisted laser desorption ionization time-of-flight (MALDI-TOF) mass spectrometry confirmed the expected correct mass for TOFYL-CoA (Figure 5(a), *m*/*z* = 1074.69) and C75-CoA (Figure 5(b), *m*/*z* = 1022.51). For each sample, the additional labeled peaks corresponded to the parent ion plus one, two, three, or four potassium ions.

*Binding of CoA Thioesters of Fatty Acid Synthesis Inhibitors to L-FABP and SCP-2: NBD-Stearic Acid Displacement Assay*

**Figure 6:**
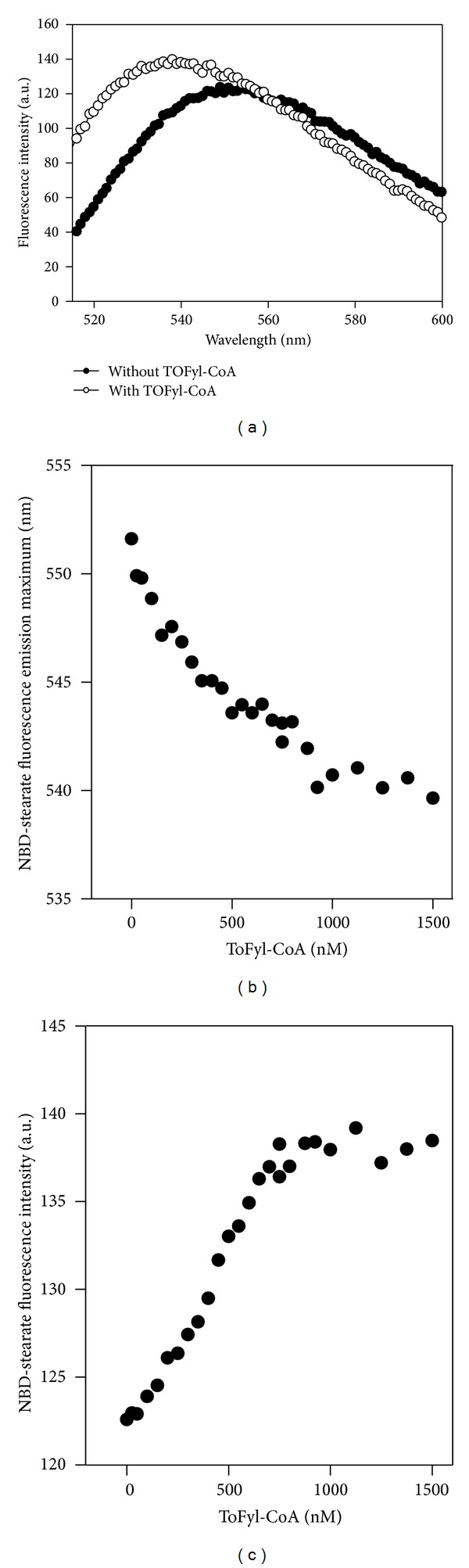
Effect of TOFyl-COA on fluorescence emission characteristics of L-FABP-bound NBD-stearic acid. NBD-stearic acid, bound to L-FABP as in Figure 3, was excited at 490 nm and fluorescence emission spectra obtained before and after addition of TOFyl-CoA (Methods). Panel (a): fluorescence emission spectra of L-FABP- (25 nM) bound NBD stearate (80 nM) without (filled circles) and with (open circles) of TOFyl-CoA (1500 nM). With increasing TOFyl-CoA concentration, the emission maximum of L-FABP-bound NBD stearate shifted to lower wavelength (panel b), and fluorescence intensity increased (panel c).

Since TOFyl-CoA did not decrease the fluorescence intensity of NBD-stearic acid bound to L-FABP, this would at first glance suggest that TOFyl-CoA did not bind to L-FABP (Figure 6(a)). Closer inspection of the L-FABP bound NBD-stearic acid emission spectra, however, revealed that TOFyl-CoA shifted the maximal emission wavelength of L-FABP-bound NBD-stearic acid from 553 to 539 nM (Figure 6(a)), suggesting that TOFyl-CoA did bind to L-FABP. This shift was confirmed by adding increasing concentrations of TOFyl-CoA, which resulted in blue shifting of the emission maximum (i.e., shorter wavelength) of L-FABP-bound NBD-stearic acid (Figure 6(b)). Concomitantly, increasing concentrations of TOFyl-CoA increased the emission intensity of NBD-stearic acid bound to L-FABP by ~15% (Figure 6(c)). It should be noted that these shifts in emission maximum and intensity were not due to TOFyl-CoA forming micelles with NBD-stearic acid since these changes were not observed in the absence of L-FABP (data not shown).

**Figure 7:**
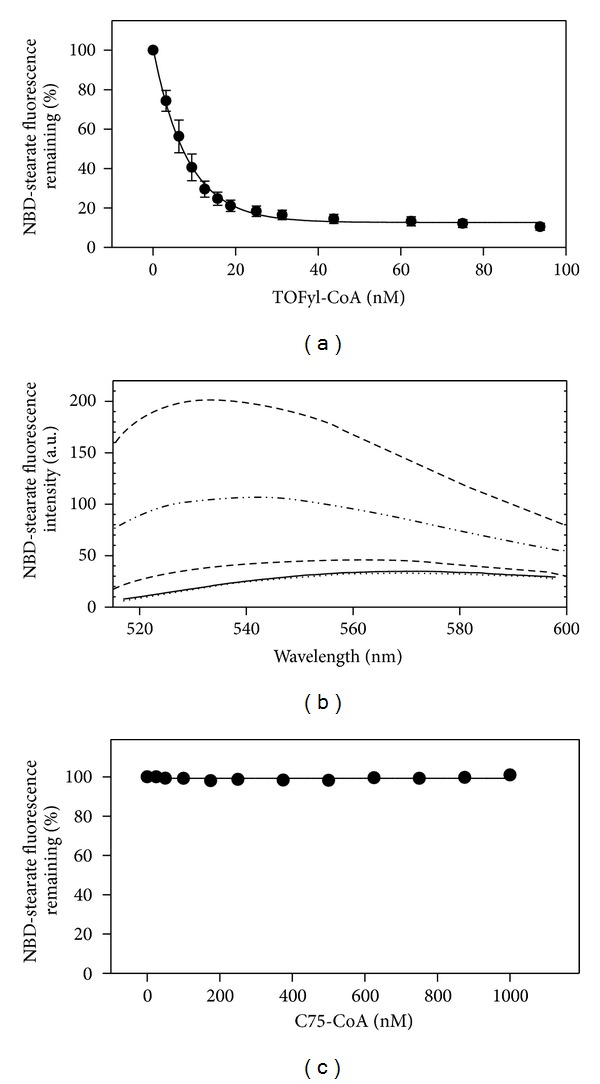
Effect of TOFyl-CoA and C75-CoA on NBD stearic binding to SCP-2. SCP-2 (25 nM in 10 mM phosphate buffer) was incubated with NBD-stearic acid (40 nM) for 12 min at 24°C to obtain maximal fluorescence. The solution was titrated with increasing amount of ligand (TOFyl-CoA or C75-CoA). TOFyl-CoA displaced SCP2-bound NBD stearate (panel a, with representative spectra in panel b). Panel (b), from top to bottom: shot dash line: NBDS+SCP2; dash-double dot-dash line: NBDS+SCP2+TOFyl-CoA (10 nM); long dashed line: NBDS+SCP2+TOFyl-CoA (100 nM); solid line: NBDS; dotted line: NBDS+TOFyl-CoA (100 nM). C75-CoA did not displace SCP2-bound NBD-stearate (panel c). Panel (a), mean ± SEM, *n* = 3.

In contrast, TOFyl-CoA completely displaced SCP-2-bound NBD-stearic acid, as shown both by the displacement curve (Figure 7(a)) and decreased emission spectra of NBD-stearic acid (Figure 7(b)). TOFyl-CoA was also very efficient in displacing SCP-2-bound NBD-stearic acid as shown by a *K*_*i*_ = 4 ± 1 nM (Table 2). In contrast, C75-CoA did not displace NBD-stearic acid from L-FABP; that is, there was no alteration in emission spectra (not shown). Likewise, C75-CoA did not displace NBD-stearic acid from SCP-2 (Figure 7(c)).

Taken together, these data suggested that TOFyl-CoA did not actually displace NBD-stearic acid from L-FABP, but by binding to L-FABP the TOFyl-CoA instead shifted NBD-stearic acid to a more hydrophobic environment within the L-FABP binding pocket. In contrast, TOFyl-CoA efficiently displaced NBD-stearic acid from SCP-2, while C75-CoA did not displace NBD-stearic acid bound to either L-FABP or SCP-2.

*Confirmation of TOFyl-CoA Binding to L-FABP*

**Figure 8:**
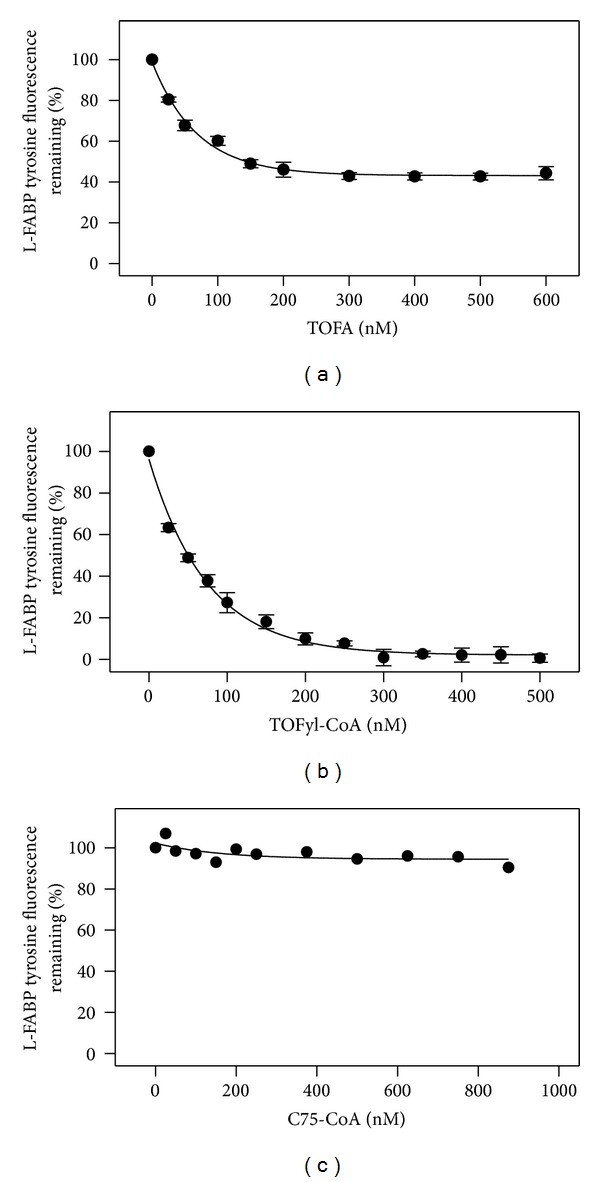
TOFA, TOFyl-CoA, and C75-CoA binding to L-FABP as determined by quenching of intrinsic L-FABP tyrosine quenching. L-FABP tyrosine quenching by TOFA (panel a), TOFyl-CoA (panel b), and C75-CoA (panel c) was determined as described in Methods. Tyrosine fluorescence emission of L-FABP (100 nM) in 10 mM phosphate buffer (pH = 7.4) was monitored by scanning from 290 to 400 nm, with excitation wavelength 280 nm, before and after small increments of added binding ligand. The binding curve was constructed by plotting percentage of tyrosine fluorescence intensity remaining at 305 nm versus concentration of the ligand. Panel (a) and (b), mean ± SEM, *n* = 3.

To further confirm that TOFyl-CoA bound to L-FABP, an intrinsic L-FABP tyrosine quenching assay was used as described in Methods. Both TOFA and TOFyl-CoA efficiently quenched L-FABP tyrosine emission (Figures 8(a) and 8(b)). Analysis of multiple L-FABP tyrosine quenching curves yielded binding affinities to L-FABP of *K*_*d*_ = 57 ± 4 nM for TOFA (Table 1) which was similar to that obtained by displacing NBD-stearic acid *K*_*d*_ = 66 ± 3 nM for TOFA (Table 1). Tyrosine quenching also determined a similar affinity of L-FABP for TOFyl-CoA as shown by *K*_*d*_ = 53 ± 4 nM (Table 2). In contrast, C75-CoA very weakly quenched L-FABP tyrosine fluorescence emission (Figure 8(c)). Very weak binding of C75-CoA to L-FABP was confirmed by the ANS displacement assay (see Methods) wherein C75-CoA displaced ANS bound to L-FABP with a *K*_*i*_ of 25.9 *μ*M (Table 2).

Taken together with the preceding data, these findings demonstrated that although L-FABP bound both fatty acid synthesis inhibitors and their CoA thioesters, these ligands were bound preferentially in the order TOFA, TOFyl-CoA > C75 > C75-CoA.

*Effect of TOFA, C75, and Their CoA Thioesters on L-FABP Secondary Structure*

Since ligand and coactivator/corepressor-induced conformational changes are a hallmark of ligand-induced nuclear receptors such as PPAR*α* [17, 27, 51, 52], the impact of the fatty acid synthesis inhibitors and their CoA thioesters on L-FABP secondary structure was determined by circular dichroism as described in Methods.

**Figure 9:**
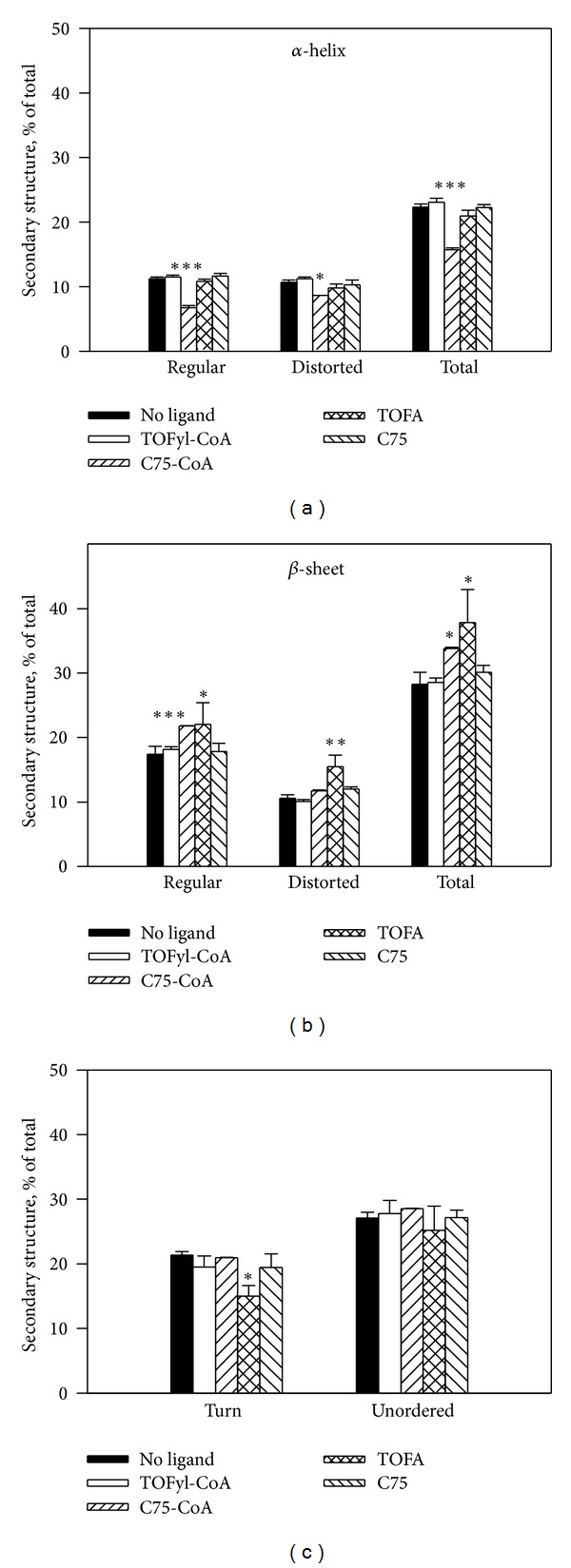
Selective fatty acid synthesis inhibitors and their CoA thioesters alter L-FABP secondary structure determined by circular dichroism (CD). L-FABP (1 *μ*M) was incubated in the absence or presence of 10 *μ*M ligand for 10 min at 25°C. Circular dichroism (CD) spectra were obtained utilizing a JASCO J-815 CD spectrometer (JASCO Analytical Instruments, Easton, MD, USA). Each sample spectrum represented the average of ten scans, and each sample spectrum was baseline corrected. Secondary structure was determined using the CONTIN algorithm as supplied by the instrument manufacturer. Statistical significance of secondary structure differences was determined by one-way ANOVA with the Newman-Keuls posttest (*n* = 3). *P < 0.05 for L-FABP + ligand versus rat L-FABP (no ligand); **P < 0.01 for rat L-FABP + ligand versus rat L-FABP (no ligand); ***P < 0.001 for rat L-FABP + ligand versus rat L-FABP (no ligand).

Although L-FABP bound both TOFA and TOFyl-CoA with high affinity (Tables 1 and 2), only TOFA significantly altered L-FABP secondary structure. While TOFA binding did not alter the proportion of *α*-helix (Figure 9(a)) or unordered structure (Figure 9(c)), the proportion of all types of *β*-sheet was increased (Figure 9(b)) concomitant with decreased turn structure (Figure 9(c)).

Likewise, while L-FABP more modestly bound C75 and C75-CoA with lower affinity than TOFA or TOFyl-CoA (Table 1), C75 did not alter L-FABP secondary structure but C75-CoA significantly altered L-FABP structure (Figure 9). In contrast, C75-CoA decreased the proportion of all types of *α*-helix (Figure 9(a)) but increased the proportion of all types of *β*-sheet (Figure 9(b)) without altering the amount of turn or unordered structures (Figure 9(c)).

Taken together these findings demonstrated that although L-FABP bound the fatty acid synthesis inhibitors and their CoA thioesters, only TOFA and C75-CoA significantly altered L-FABP's secondary structure.

*Mouse Primary Hepatocytes as a Model for Examining the Impact of LCFA Synthesis Inhibitors and L-FABP on PPAR*α* Transcriptional Activity*

**Figure 10:**
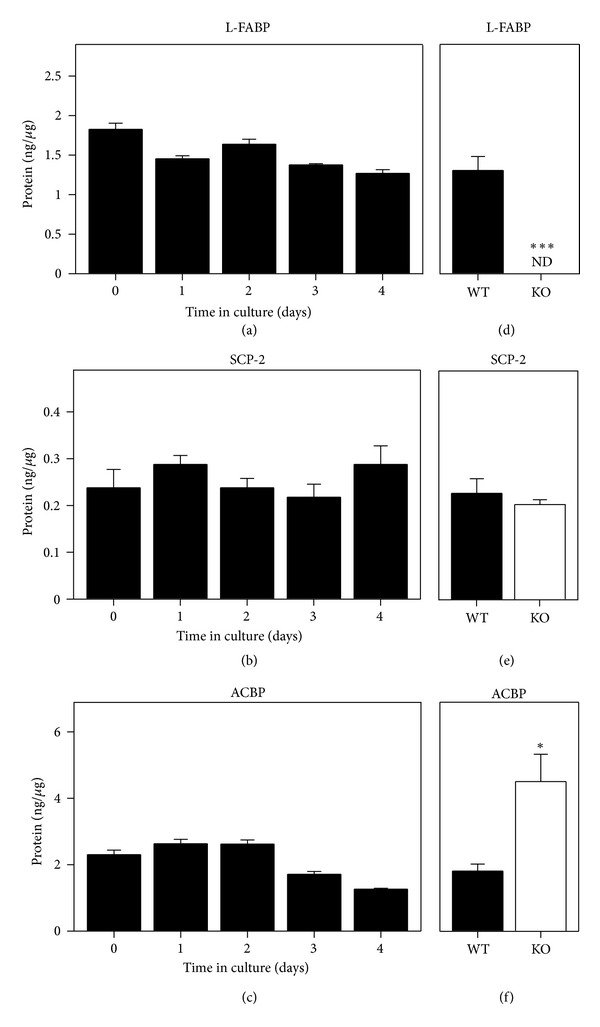
Expression of LCFA and LCFA-CoA binding proteins in cultured mouse primary hepatocytes. Primary hepatocytes from wild-type and L-FABP null mice were isolated from mouse livers and maintained in culture for up to four days as described in Methods. Quantitative western blotting was performed by comparison to a standard curve of known amounts of the respective recombinant L-FABP, SCP-2, or ACBP on the same blot as described in Methods. Quantitative western blots were obtained as a function of increasing time for wild-type hepatocytes in culture: (a) L-FABP; (b) SCP-2; and (c) ACBP. Time 0 = concentration in liver while time of 1–4 days indicates time in culture. For determining the effect of L-FABP gene ablation on expression of these proteins, quantitative western blots of (d) L-FABP; (e) SCP-2; and (f) ACBP were also obtained for hepatocytes from wild-type (WT) and L-FABP null (KO) hepatocytes after three days in culture. Mean ± SEM, *n* = 3–6.

**Figure 11:**
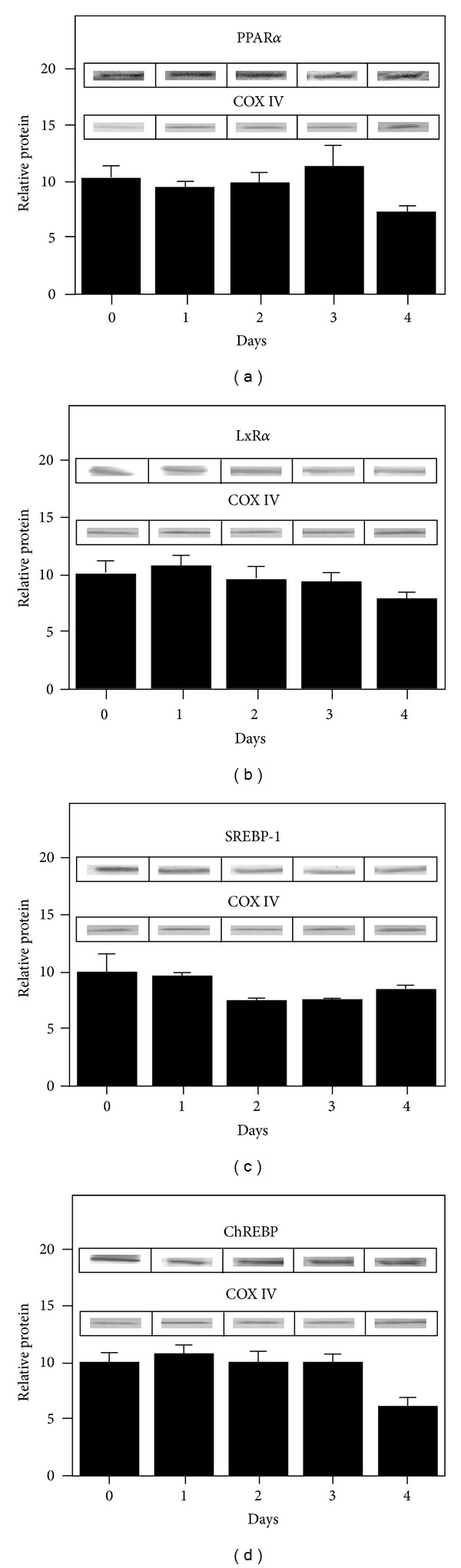
Expression of key nuclear receptors involved in fatty acid and glucose metabolism as a function of time. Primary hepatocytes were isolated from mouse livers and maintained in culture for up to four days as described in Methods. Representative western blots relative to a housekeeping protein (COX-1) are shown in the inserts. Quantitative analysis of multiple western blots relative to housekeeping protein was shown as black bars for PPAR*α* (Panel a), LXR*α* (Panel b), SREBP1 (Panel c), and ChREBP (Panel d) as described in Methods. Time 0 = concentration in liver while time of 1–4 days indicates time in culture. Mean ± SEM, *n* = 3–6.

While there are limitations to any *in vitro* model, cultured primary mouse hepatocytes are a physiologically relevant and more controlled system without competition by other tissues for LCFA synthesis inhibitors, organ-specific cross talk, or endocrine influences [53]. Our labs previously established that primary mouse hepatocytes maintained expression of key proteins, enzymes, and receptors involved in the uptake of LCFAs (FATP5, GOT, FATP2, FATP4) and glucose (GLUT2, GLUT1, glucokinase, insulin receptor) similar to those in liver for 2-3 days in culture [43, 54–56]. Mouse primary hepatocytes also maintained expression of cytosolic LCFA/LCFA-CoA binding/transport proteins including L-FABP (Figure 10(a)), SCP-2 (Figure 10(b)), and ACBP (Figure 10(c)). Finally, mouse primary hepatocytes' expression of PPAR*α* (Figure 11(a)), LXR*α* (Figure 11(b)), and CHREBP (Figure 11(c)) was also similar to liver for 3 days in culture. SREBP-1 expression was the same as liver and constant for 1 day, decreasing slightly thereafter (Figure 11(c)). Thus, for all subsequent studies of fatty acid synthesis inhibitor effects (in the context of the presence or absence of L-FABP) on PPAR*α* transcriptional activity of fatty acid *β*-oxidative enzymes the mouse primary hepatocytes were cultured ≤2 days.

*Impact of Fatty Acid Synthesis Inhibitors on PPAR*α* Transcription of Mitochondrial (CPT1A, CPT2) and Peroxisomal (ACOX1) Fatty Acid *β*-Oxidative Enzymes in Wild-Type Mouse Primary Hepatocytes: Role of Glucose*

**Figure 12:**
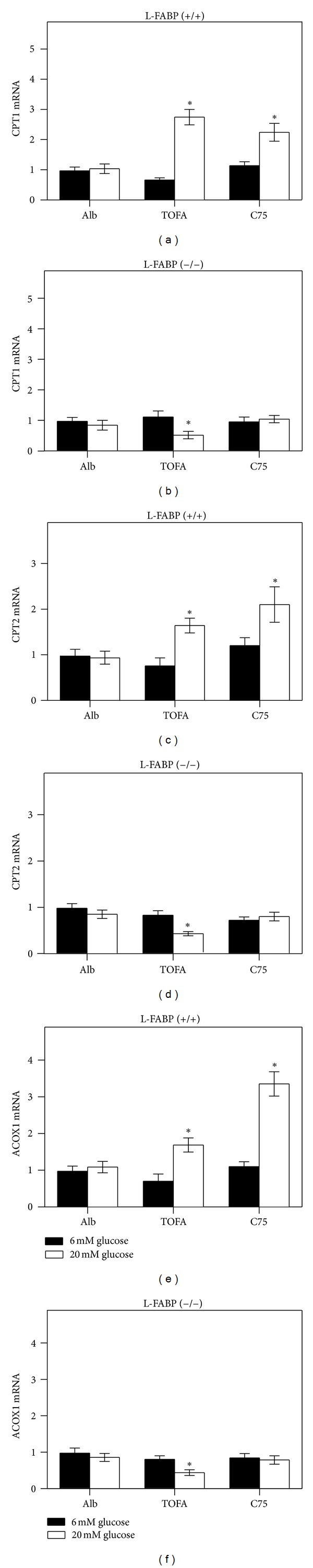
Effect of TOFA and C75 on CPT1, CPT2, and ACOX1 gene expression in cultured mouse primary hepatocytes isolated from livers of wild-type (WT, L-FABP (+/+)) and null [(L-FABP (−/−)] mice. Hepatocytes isolated from wild-type [WT, L-FABP (+/+)] or gene-ablated [null, L-FABP (−/−)] mice were preincubated for 30 min with 10 *μ*g/mL TOFA or C75 in serum-free culture medium before addition of glucose (6 or 20 mM) as described in Methods. Total RNA was isolated from hepatocytes 5 hr after glucose addition and used for quantitative real-time PCR. The fold change in CPT1A (a, b), CPT2 (c, d), ACOX1 (e, f) mRNA levels was determined relative to internal control housekeeping gene as described in Methods. Values for each genotype were expressed relative to [Alb + 6 mM glucose] within that genotype. Panels (a), (b): CPT1A mRNA fold changes in WT and L-FABP null hepatocytes; (c), (d): CPT2 mRNA fold changes in WT and L-FABP null hepatocytes; (e), (f): ACOX1 mRNA fold changes in WT and L-FABP null hepatocytes. Mean ± SEM, *n* = 3.

When hepatocytes were cultured in medium with normal physiological glucose (6 mM), neither TOFA nor C75 significantly altered PPAR*α* transcription of CPT1A (Figure 12(a), black bars), CPT2 (Figure 12(c), black bars), or ACOX1 (Figure 12(e), black bars). In contrast, at high (20 mM) glucose in the culture medium both TOFA and C75 induced PPAR*α* transcription of CPT1A 2 (Figure 12(a), open bars), CPT2 (Figure 12(c), open bars), and ACOX1 (Figure 12(e), open bars). Thus, high glucose conferred on TOFA and C75 the ability to induce PPAR*α* transcriptional activity.

*Fatty Acid Synthesis Inhibitors Induce PPAR*α* Transcription of Mitochondrial (CPT1A, CPT2) and Peroxisomal (ACOX1) Fatty Acid *β*-Oxidative Genes in Primary Hepatocytes from Mice: Role of L-FABP*

At physiologically normal glucose (6 mM) in the culture medium, L-FABP gene ablation did not alter the lack of effect of TOFA or C75 on PPAR*α* transcription of CPT1A (Figure 12(b), black bars), CPT2 (Figure 12(d), black bars), or ACOX1 (Figure 12(f), black bars). However, at high glucose (20 mM) L-FABP gene ablation abolished the ability of TOFA and C75 to activate PPAR*α* transcription (Figures 12(b), 12(d), and 12(f), open bars). On the contrary, at 20 mM glucose and in the presence of TOFA the loss of L-FABP decreased by nearly 50% the PPAR*α* transcription of CPT1A (Figure 12(b), open bars), CPT2 (Figure 12(d), open bars), or ACOX1 (Figure 12(f), open bars).

This loss of the ability of fatty acid synthesis inhibitors to induce PPAR*α* transcriptional activity in the context of high glucose was associated with complete loss of L-FABP (Figure 10(d)). L-FABP gene ablation did not downregulate the level of the other LCFA/LCFA-CoA binding proteins, SCP-2 (Figure 10(e)), and ACBP (Figure 10(f)). Instead, the level of SCP-2 was unchanged (Figure 10(e)) while that of ACBP was actually upregulated (Figure 10(f)) in L-FABP null hepatocytes.

*Effect of High Glucose in the Culture Medium on Cytosolic Glucose: Impact of Fatty Acid Synthesis Inhibitors*

High glucose enhances L-FABP interaction with PPAR*α*, and nucleoplasmic glucose levels are similar to cytoplasmic [49, 57]. Therefore, the possibility that high glucose in the culture medium raised intracellular glucose levels was examined in the absence or presence fatty acid synthesis inhibitors.

**Figure 13:**
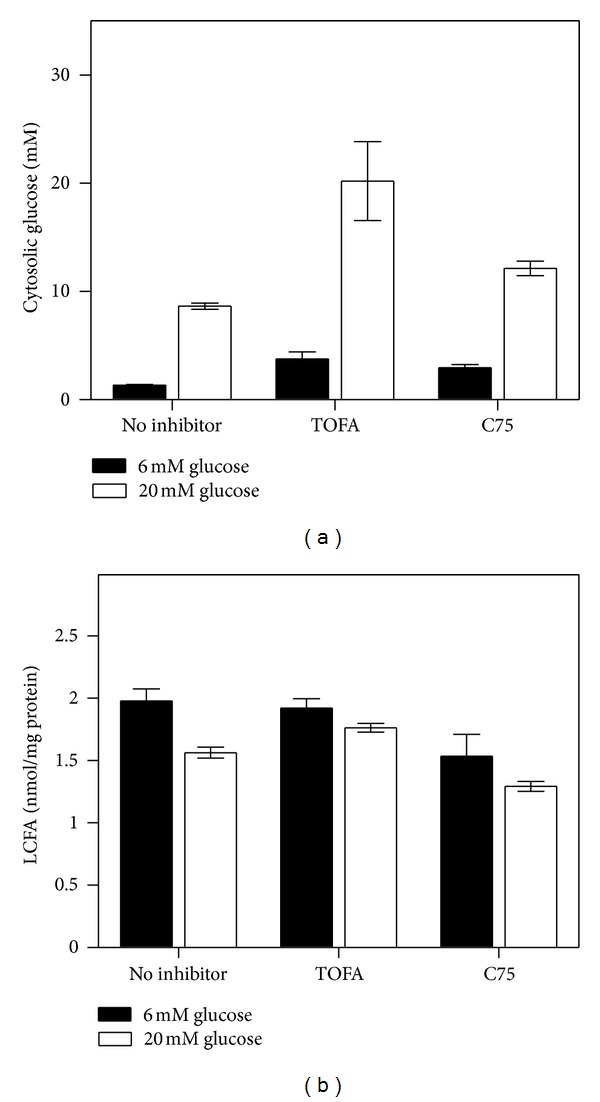
Cytosolic glucose (panel a) and free fatty acid (LCFA, panel b) levels in mouse primary hepatocytes cultured with and without TOFA or C75. Hepatocytes were incubated with TOFA or C75 (10 *μ*g/mL) with 6 or 20 mM glucose (Section 2). Cytosolic glucose level was then determined as in Methods. Briefly, hepatocytes were washed quickly with ice old solution of MgCl_2_ (100 mM) with 0.1 mM phloretin. Cells were then homogenized with a probe sonicator and, after centrifugation, the supernatant was used for glucose analysis with the Amplite Glucose Quantitation Kit as instructed by the manufacturer. For LCFA determination, hepatocyte homogenate was extracted twice with 1% Triton X-100 in pure chloroform. The organic phase was collected, and the FFA content of each sample was measured with the Free Fatty Acid Quantification Kit from BioVision, Inc. according to manufacturer's instructions using enclosed palmitic acid as standard. Mean ± SEM, *n* = 3.

When mouse primary hepatocytes were cultured in medium without fatty acid synthesis inhibitors but with normal physiological (6 mM) glucose, cytosolic glucose was near 2 mM (Figure 13(a), black bar, no inhibitor). Increasing glucose level from 6 to 20 mM, in the absence of fatty acid synthesis inhibitors, increased cytosolic glucose in parallel by nearly 4-fold to 9 mM (Figure 13(a), no inhibitor).

Interestingly, in the presence of TOFA or C75 in the culture medium containing normal physiological (6 mM) glucose, mouse primary hepatocyte cytosolic glucose was increased ~2-fold to 4 mM (Figure 13(a), black bars, TOFA or C75). In the presence of TOFA or C75 with increasing glucose (from 6 to 20 mM) the cytosolic glucose was increased in parallel by nearly 5- and 4-fold to 20 and 13 mM, respectively, (Figure 13(a), open bars, TOFA or C75).

Taken together, these data showed that high glucose (20 mM) alone or fatty acid synthesis inhibitors (TOFA or C75) alone increased cytoplasmic glucose to about 45% or 80% compared to that of extracellular glucose. High glucose together with TOFA or C75 increased cytoplasmic glucose even more such that it was nearly the same as that in the culture medium. Thus, cultured primary hepatocyte cytosolic glucose level was about half of that in the culture medium but highly responsive to the extracellular glucose concentration. Further, fatty acid synthesis inhibitors (TOFA, C75) precluded incorporation of glucose-derived acetyl CoAs into fatty acids, and thus intracellular glucose increased to near extracellular levels.

*Fatty Acid Synthesis Inhibitors Had Little Effect on Cellular Mass of Unesterified Fatty Acids*

Although the majority (>93%) of LCFAs synthesized *de novo* from glucose are saturated (C14 : 0, C16 : 0, C18 : 0) [58] and do not bind to PPAR*α* [19, 20, 24], the remaining 7% (primarily C18 : 1n-9 and C18 : 2n-6) are bound and/or weakly activate PPAR*α* [20, 59–63]. Although monounsaturated LCFAs such as 18:1n-9 have little effect on PPAR*α* activity or PPAR*α*-regulated genes in cultured primary hepatocytes [64], a previous study suggested that *de novo* synthesized endogenous LCFA may also be PPAR*α* agonists in liver as demonstrated in fatty-acid synthase knockout in liver of mice [65]. Therefore, the possibility that inhibition of *de novo* fatty acid synthesis (especially by TOFA) at high glucose led to the decreased cellular levels of unesterified fatty acids was examined. Neither TOFA nor C75 significantly impacted the unesterified fatty acid level in mouse primary hepatocytes cultured with normal physiological glucose in the medium (Figure 13(b), black bars). High glucose (20 mM) alone decreased cellular unesterified fatty acid level slightly, an effect not further exacerbated by *de novo* fatty acid synthesis inhibitors (Figure 13(b), open bars). Thus, overall the inhibitors of *de novo* fatty acid synthesis did not significantly alter hepatocyte levels of unesterified fatty acids. The maintenance of a near constant level of unesterified fatty acid was likely due to the presence of exogenous fatty acids taken up from the medium and/or to release of fatty acids from intracellular lipid storage droplets.

## 4. Discussion

To help explain some of the paradoxical findings involving fatty acid synthesis inhibitors, we hypothesized, based on their structural similarity to fatty acids, that they may mediate part of their action through the L-FABP signaling to PPAR*α* in the nucleus. Experimental findings presented herein make the following significant contributions to our understanding of the mechanism(s), whereby the fatty acid synthesis inhibitors may also act by affecting PPAR*α*-regulated expression of fatty acid oxidative enzymes in liver.

First, L-FABP bound fatty acid synthesis inhibitor TOFA and its thioester TOFyl-CoA. L-FABP affinities for TOFA and TOFyl-CoA were in the same range as those for potent PPAR*α* activators such as n-3 polyunsaturated fatty acids and fenofibrate [30, 31, 66–69]. It is important to note, however, that TOFA and TOFyl-CoA bound L-FABP in a manner similar to, but also somewhat different, from that of analogous chain length natural LCFAs, LCFA-CoAs, and LCFA derivatives (HETEs, prostaglandins) and a variety of peroxisome proliferator agents (fibrates, fibroyl-CoAs, eicosatetraynoic acid, WY14,643, BRL48,482) [18, 66–68, 70–72]. Our finding of two NBD-stearic acid binding sites on L-FABP was consistent with most other fluorescence, isothermal titration microcalorimetry, and NMR studies, which also detected two-LCFA or LCFA-CoA binding sites on L-FABP [18, 66, 68, 71–73]. However, our finding that both TOFA and TOFyl-CoA bound to L-FABP but only TOFA displaced L-FABP-bound NBD-stearic acid suggests the presence of an additional site on L-FABP that bound TOFyl-CoA but not NBD-stearic acid. Consistent with this possibility, radioligand binding, NMR, and X-ray crystallography all detect the presence of additional site(s) that binds LCFA or smaller molecules [74–79].

Second, L-FABP also bound the fatty acid synthesis inhibitor C75 and its thioester C75-CoA but more weakly than TOFA or its thioester. C75 and C75-CoA were less able to displace bound NBD-stearic acid or ANS. This finding was consistent with L-FABP less strongly binding 11-12 atom chain-length fatty acids and/or their CoA thioesters than their longer chain counterparts [80, 81]. This suggested that perhaps C75 and/or C75-CoA might also interact with the previously mentioned additional binding site on L-FABP without altering NBD-stearic acid binding or L-FABP tyrosine emission [72, 82]. Earlier studies suggested the presence of an additional ligand binding site in L-FABP [74–79]. Regardless, however, it is important to note that while L-FABP's affinity for C75 and C75-CoA was significantly lower than that of TOFA and its thioesters, nevertheless these affinities were in the range of the less potent fibrate activators of PPAR*α* such as bezafibrate and clofibrate [30, 31, 33, 69].

Third, L-FABP not only binds the fatty acid synthesis inhibitors, but several (TOFA, C75-CoA) also altered L-FABP's secondary structure, suggesting that this in turn may facilitate L-FABP ligand signaling to PPAR*α*. In support of this possibility L-FABP directly binds to PPAR*α* [17, 33, 49, 83]. Further, fatty acid or fibrate binding alters L-FABP structure to stabilize the ligand portal region of L-FABP for directly channeling bound ligands for optimal transfer to PPAR*α* [17, 33, 49, 83, 84]. Although it has been speculated that PPAR*α* may bind TOFA, to date there have been no reports determining the possibility that TOFA, C75, or their CoA thioesters bind PPAR*α*. However, an examination of their structures (Figure 1) shows that these ligands contain C10 to C14 carbon chains. Radioligand binding assays reveal that 10–14 carbon fatty acids are bound by PPAR*α* (and more weakly by other PPARs) and activate PPAR*γ* [85, 86]. On this basis, it can be hypothesized that TOFA, C57, and/or their respective CoA derivatives may also bind to PPAR*α* such that induction was significantly potentiated by high glucose in the culture medium, albeit requiring L-FABP.

Fourth, TOFA and C75 induced PPAR*α* transcription of mitochondrial (CPT1A, CPT2) and peroxisomal (ACOX1) fatty acid *β*-oxidative enzymes in cultured primary mouse hepatocytes. Thus, TOFA stimulated CPT1 activity and fatty acid oxidation not only by reducing malonyl-CoA levels as suggested earlier [10], but also by inducing PPAR*α* transcription of CPT1 as well as other LCFA *β*-oxidative enzymes. In support of this finding in mouse hepatocytes, TOFA also transactivated PPAR*α* in COS7 cells and increased expression of PPAR*α* itself regulated by PPAR*α* ligands [87, 88]. Likewise, C75 stimulated CPT1A activity and fatty acid oxidation not only by binding to CPT1 to prevent inhibition by malonyl CoA or by reducing ACC expression as hypothesized earlier [3, 13], but also by inducing PPAR*α* transcription of CPT1A and other LCFA *β*-oxidative enzymes (CPT2, ACOX1) in the nucleus. In contrast to TOFA and C75, cerulenin has only a single carbonyl group, is not amphipathic, does not increase CPT1 activity, and does not transactivate PPAR*α* in COS7 cells [13, 88]. Taken together, these findings suggest that fatty acid synthesis inhibitors such as TOFA and C75 may exert their lipid lowering effects at least in part by inducing PPAR*α* transcription of CPT1A and other fatty acid *β*-oxidative enzymes in tissues such as liver.

Fifth, TOFA and C75 induced PPAR*α* transcription of fatty acid *β*-oxidative enzymes only when the mouse primary hepatocytes were cultured in the presence of high glucose in the medium. In support of this finding, previous studies have demonstrated that TOFA and C75 increased CPT1A activity and fatty acid oxidation in rat hepatocytes cultured in media containing high glucose (11 mM) [10, 12–14]. Interestingly, a variety of other LCFAs (PUFA > MUFA > saturated) and xenobiotics (fibrates, Wy-14643) activate PPAR*α* transcription when hepatocytes were cultured in medium containing high levels of glucose (11–28 mM) [44, 89–97]. Unfortunately, the effects of these L-FABP ligands at normal physiological (6 mM) glucose were not reported. Although TOFA transactivated PPAR*α* in COS7 cells cultured in commercially available DMEM medium [88], it is not clear which of the available DMEM formulations (i.e., 25, 5.6, or 0 mM glucose) was used. However, studies from our laboratory have shown that other lipidic ligands (arachidonic acid, clofibrate) transactivate PPAR*α* much more with increasing glucose in DMEM culture medium [98]. It is important to note that the potentiation of TOFA and C75 induction of PPAR*α* transcription of fatty acid *β*-oxidative enzymes at high glucose correlated with high cytosolic glucose. Cytosolic glucose is >100-fold lower than outside in most peripheral cells [46, 98–100], but liver cytosolic glucose is much higher (~4 mM) [101, page 59], [102, 103], consistent with the 2 mM glucose in hepatocytes cultured with normal physiological glucose (6 mM) shown herein. The cytosolic glucose concentration in hepatocytes higher than most other cell types is due to the presence of a higher *K*_*m*_ glucose transporter (GLUT2), a higher *K*_*m*_ hexokinase (glucokinase), different insulin sensitivity, and different metabolic activity [101, page 59], [102, 103]. High glucose in the medium (20 mM) significantly increased cytoplasmic glucose to ~9 mM. Potentiation of TOFA and C75 induction of PPAR*α* transcription at high extracellular glucose (20 mM) correlated with TOFA and C75 both increasing cytosolic glucose nearly by 2-fold. In an earlier study, TOFA treatment of rat primary hepatocytes cultured with Krebs-Henseleit medium (11 mM glucose) induced glucose accumulation and release into the medium [10]. This finding was attributed to TOFA inhibiting glycolysis, likely as a consequence of accelerated fatty acid oxidation, which in turn decreased the rate of net glucose and glycogen utilization [10]. Finally, the effects of high glucose in conferring on fatty acid synthesis inhibitors the ability to induce PPAR*α* transcriptional activity were not likely mediated through glucose-induced posttranslational modification of L-FABP or PPAR*α*, such as through phosphorylation or sumoylation. L-FABP does not appear to be modified by these processes [70, 104, 105]. Although insulin induces phosphorylation of PPAR*α* to activate its transcriptional activity [106, 107], in the study presented herein insulin was maintained at a constant level in the culture medium. In addition, high glucose without inhibitor did not induce PPAR*α* transcription of CPT1A, CPT2, or ACOX1. Likewise, while PPAR*α* sumoylation represses PPAR*α* activity [108], our studies indicate that high glucose increased rather than decreased PPAR*α* transcriptional activity. It has been shown that hyperglycemic conditions increase intracellular glucose in a variety of primary (human endothelial cells, bovine retinal pericytes) and established (fibroblasts, COS7 cells) cell lines [98, 109]. The studies presented herein demonstrate that high glucose in the culture medium also increases intracellular glucose in cultured primary mouse hepatocytes. While it is not known if hepatic glucose is elevated in diabetes, several studies have shown that diabetes increased intracellular glucose in muscle and retinal cells [46, 99].

Sixth, L-FABP contributed significantly to TOFA and C75 induction of PPAR*α* transcription in the context of high glucose in the mouse primary hepatocyte culture medium. L-FABP provides a signaling pathway for an analogous broad variety of lipidic ligands (straight and branched chain LCFAs, LCFA-CoAs, fibrates, and xenobiotics), chaperoning them to the nucleus [17–20]. L-FABP directly interacts with PPAR*α* in the nucleus, and this binding is enhanced at high glucose (review in [17, 18, 49, 83]). L-FABP gene ablation abolished the ability of TOFA as well as C75 to stimulate PPAR*α* transcription of CPT1, CPT2, and ACOX1 in hepatocytes cultured with high glucose, apparently by different but overlapping mechanisms. Thus, our findings indicate that TOFA and C75 stimulating PPAR*α* transcription of LCFA *β*-oxidative enzymes (CPT1A, CPT2, ACOX1) at high glucose were mediated through L-FABP. Earlier studies from our and other laboratories showed that glucose binds to both L-FABP and PPAR*α* to alter their conformations and high glucose increases L-FABP's binding affinity for PPAR*α* [83, 98, 110, 111].

In summary, the fatty acid synthesis pathway has become a therapeutic target for ameliorating the adverse effects of obesity as well as its associated type 2 diabetes and cardiovascular disease. Drugs such as TOFA and C75 target the fatty acid metabolic pathway to inhibit synthesis, thereby decreasing body weight, adipose tissue, hyperlipidemia, and fatty liver. While initially thought of primarily as inhibitors of *de novo* fatty acid synthesis, these agents also exhibit additional effects in the central sympathetic nervous system (decrease food intake) and liver (increase CPT1 activity and fatty acid oxidation) [8–11]. The effects of TOFA and C75 on CPT1A are paradoxical, explained only in part by their impact on the level of malonyl-CoA and/or direct interaction with the CPT1 enzyme. The results presented herein demonstrate that TOFA and C75 can also induce PPAR*α* transcription. This induction was significantly potentiated by high glucose in the culture medium and required L-FABP. L-FABP bound TOFA at classic LCFA binding site(s) on L-FABP, while TOFyl-CoA and likely C75 and/or C75-CoA interact through an additional site(s). Within the cell, L-FABP enhances LCFA uptake, transport through the cytosol, and provides a signaling pathway for bound ligands into the nucleus, where L-FABP directly binds to PPAR*α* to deliver the bound ligand (review in [17, 18, 49]). Taken together with the data presented herein, these findings delineate a *novel* mechanism whereby high glucose enables *de novo* LCFA synthesis inhibitors to enhance LCFA oxidation through PPAR*α*, similarly as demonstrated with natural LCFAs. It is known that the adverse effects of chronic hyperglycemia in human subjects are exacerbated by high dietary fat rich in saturated LCFAs, poor ligands and activators of PPAR*α* [19, 20, 24, 112, 113]. In contrast, hypolipidemic drugs such as fibrates that are more potent PPAR*α* agonists appear more effective in subjects with hyperglycemia such as in type 2 diabetes than in nondiabetic dyslipidemics [114]. It is thus interesting to speculate that higher glucose levels in poorly controlled diabetics may also positively impact fatty acid synthesis inhibitor activation of PPAR*α* transcriptional activity as compared to the normoglycemic population.

## Figures and Tables

**Table 1:** Binding constants of fatty acids, TOFA, and C75 to L-FABP and SCP-2.

LIGAND	Binding constants (*μ*M)	
	L-FABP	SCP2
Fluorescent fatty acids		
NBD stearate	*K*_*d*1_ = 0.018 ± 0.001 *K*_*d*2_ = 0.055 ± 0.002	0.101 ± 0.003

Endogenous fatty acid		
Stearic acid	0.087 ± 0.006^(a)^	ND

Fatty acid synthesis inhibitors		
TOFA	0.066 ± 0.003^(a)^ 0.057 ± 0.004^(c)^	0.227 ± 0.020^(a)^
C75	5.59 ± 0.31^(b)^	not bound^(a)^

*K*_*i*_ was determined by displacing protein-bound NBD-stearic acid (a) or ANS (b) as in Methods. (c), *K*_*d*_ was determined by L-FABP tyrosine quenching. ND: not determined. Values are the mean ± SEM, *n* = 3.

**Table 2:** Binding parameters for L-FABP and SCP2 to CoA interacting with fatty acid and fatty acid synthesis inhibitor CoA thioesters.

	Binding constants (*μ*M)	
	L-FABP	SCP2
Stearoyl-CoA	0.46 ± 0.03^(a)^	ND
TOFyl-CoA	0.053 ± 0.004^(c)^	0.004 ± 0.001^(a)^
C75-CoA	25.9 ± 3.9^(b)^	Not bound^(a)^

*K*_*i*_ was determined by displacing protein-bound NBD-stearic acid (a) or ANS (b) or by intrinsic L-FABP tyrosine quenching (c) as in Methods. ND: not determined. Values are the mean ± SEM, *n* = 3.

## Abbreviations

AA: Arachidonic acid, C20 : 4, n-6
ACAC: Acetyl CoA carboxylase
ACOX1: Acyl-CoA oxidase 1, palmitoyl
ANS: Aminonaptholsulfonic acid
CPT1A: Carnitine palmitoyl transferase I, liver
CPT2: Carnitine palmitoyl-CoA transferase II
C75: 4-methylene-2-octyl-5-oxotetrahydrofuran-3-carboxylic acid
cerulenin: ([2S,3R]2,3-epoxy-4-oxo-7E,10E-dodecadienamide)
DHA: Docosahexaenoic acid, C22 : 6, n-3
EPA: Eicosapentaenoic acid, C20 : 5, n-3
FASN: Fatty acid synthase
LCFA: Long chain fatty acids, unesterified
LCFA-CoA: Long chain fatty acyl CoA
L-FABP: Liver fatty acid binding protein
L-FABP(−/−): L-FABP knock out mouse genotype
L-FABP(+/+): Wild-type mouse genotype
PPPA*α*: Peroxisome proliferator activated receptor-*α*
NBD-stearate: 12-(N-methyl)-N-[(7-nitrobenz-2-oxa-1,3-diazol-4-yl)-amino]-octadecanoic acid
PBS: Phosphate buffered saline, pH 7.4
PUFA: Polyunsaturated fatty acid, very long chain, C20–C22
SCP-2: Sterol carrier protein-2
TOFA: 5-tetradecyloxy-2-furancarboxylic acid.
